# Targeting NRF2-Governed Glutathione Synthesis for *SDHB*-Mutated Pheochromocytoma and Paraganglioma

**DOI:** 10.3390/cancers12020280

**Published:** 2020-01-23

**Authors:** Yang Liu, Ying Pang, Veronika Caisova, Jianyi Ding, Di Yu, Yiqiang Zhou, Thanh-Truc Huynh, Hans Ghayee, Karel Pacak, Chunzhang Yang

**Affiliations:** 1 Neuro-Oncology Branch Center for Cancer Research, National Cancer Institute, Bethesda, MD 20892, USA; yang.liu5@nih.gov (Y.L.); jianyi.ding@nih.gov (J.D.); yudi_1808@dicp.ac.cn (D.Y.); zhou.yiqiang@outlook.com (Y.Z.); 2 Section on Medical Neuroendocrinology, *Eunice Kennedy Shriver* National Institute of Child Health and Human Development, National Institutes of Health, Bethesda, MD 20892, USA; ying.pang@nih.gov (Y.P.); caisovav@gmail.com (V.C.); huynht@mail.nih.gov (T.-T.H.); 3 Division of Endocrinology, Department of Medicine, University of Florida, Gainesville, FL 32610, USA; hans.ghayee@medicine.ufl.edu; 4 Malcom Randall VA Medical Center, Gainesville, FL 32608, USA

**Keywords:** NRF2, glutathione metabolism, SDHB mutation, pheochromocytoma, paraganglioma

## Abstract

Succinate dehydrogenase subunit B (SDHB) deficiency frequently occurs in cluster I pheochromocytomas and paragangliomas (PCPGs). *SDHB*-mutated PCPGs are characterized by alterations in the electron transport chain, metabolic reprogramming of the tricarboxylic cycle, and elevated levels of reactive oxygen species (ROS). We discovered that *SDHB*-deficient PCPG cells exhibit increased oxidative stress burden, which leads to elevated demands for glutathione metabolism. Mechanistically, nuclear factor erythroid 2-related factor 2 (NRF2)-guided glutathione de novo synthesis plays a key role in supporting cellular survival and the proliferation of SDHB-knockdown (*SDHB^KD^*) cells. NRF2 blockade not only disrupted ROS homeostasis in *SDHB*-deficient cells but also caused severe cytotoxicity by the accumulation of DNA oxidative damage. Brusatol, a potent NRF2 inhibitor, showed a promising effect in suppressing *SDHB^KD^* metastatic lesions in vivo, with prolonged overall survival in mice bearing PCPG allografts. Our findings highlight a novel therapeutic strategy of targeting the NRF2-driven glutathione metabolic pathway against *SDHB*-mutated PCPG.

## 1. Introduction

Pheochromocytomas and paragangliomas (PCPGs) are neuroendocrine tumors derived from chromaffin cells, which are commonly located in adrenal and extra-adrenal compartments. Genetically, PCPG tumorigenesis is related to genetic alterations in 21 genes, which cluster into three major molecular subtypes on the basis of their signature transcriptomic profiles [1,2,3]. Cluster I PCPGs are characterized by the activation of the pseudohypoxia-related signaling pathway, which includes mutations in hypoxia-inducible factor 2A (*HIF2A*), succinate dehydrogenase subunits (*SDHA*, *SDHB*, *SDHC*, *SDHD*), succinate dehydrogenase complex assembly factor 2 (*SDHAF2*), von Hippel–Lindau tumor suppressor (*VHL*), egl-9 prolyl hydroxylases 1 and 2 (*EGLN1/2*), fumarate hydratase (*FH*), malate dehydrogenase 2 (*MDH2*), and the ATP-dependent helicase (*ATRX*). Meanwhile, cluster II PCPGs result from mutations related to the kinase signaling pathway, which includes the *RET* proto-oncogene, neurofibromin 1 (*NF1*), Harvey rat sarcoma proto-oncogenes (*H-RAS*), transmembrane protein 127 (*TMEM127*), and Myc-associated factor X (*MAX*) [2,4,5,6,7,8,9,10]. Cluster III PCPGs are a recently identified disease subtype, with a transcriptomic signature of Wnt/β-catenin signaling [8]. Among all molecular subtypes of PCPGs, genetic abnormalities in *SDHx* underlie the most aggressive phenotype, with a strong tendency to metastatic disease, tumor multiplicity, and recurrence [11,12,13,14].

The current standards of care for metastatic PCPGs usually provide marginal benefit to tumor suppression or elimination and disease outcome including survival. In terms of chemotherapy, a combination regimen including cyclophosphamide, vincristine, and dacarbazine (CVD) is currently recommended as the first-line therapy to manage rapidly progressing metastatic PCPGs, whereas the responses are usually transient [15]. Several longitudinal studies have revealed that although the CVD regimen provides objective tumor responses, it fails to yield improvements in overall survival [16,17]. On the other hand, some recent studies have shown that the distinctive transcriptional profile in cluster I PCPG may give rise to a distinctive spectrum of therapeutic options. For example, Hadoux et al. discovered that temozolomide was more effective against metastatic *SDHB* PCPG due to low O^6^-alkylguanine DNA alkyltransferase (MGMT) methylation status [18]. In addition, Sulkowski, as well as our group, discovered that *SDHB* PCPGs exhibit higher sensitivity to a combination regimen involving a poly (adenosine diphosphate-ribose) polymerase (PARP) inhibitor and genotoxic agent, as this type of malignancy exhibits a deficiency of homologous recombination DNA repair and nicotinamide adenine dinucleotide (NAD) metabolism [19,20]. Overall, these findings imply that therapeutic regimens can be optimized by targeting the unique molecular signature(s) of cluster I PCPGs.

Nuclear factor (erythroid-derived 2)-like 2 (*NFE2L2*; NRF2) is a transcriptional factor that governs cellular redox homeostasis by mediating the trans-activation of antioxidant-related genes. Oxidative stress has been recognized as a hallmark of cancers, which promotes tumor growth and malignant progression, especially for cancers with intrinsic altered metabolic signatures, such as *SDHB*-mutated PCPGs and isocitrate dehydrogenase 1 (*IDH1*)-mutated malignancies. These cancers tend to develop a dependency on reactive oxygen species (ROS) detoxification pathways to maintain a reasonable ROS level [21]. The distinctive role of the antioxidant pathway in these malignancies suggests that targeting ROS-scavenging pathways could be a valuable anticancer strategy. Owing to the close relevance to therapeutic resistance and detoxification pathways, targeting NRF2 has long been proposed as a potential cancer therapeutic approach [22,23]. Meanwhile, pioneering work by Ren et al. suggested that brusatol, a plant-derived natural quassinoid, serves as a potent inhibitor of the NRF2 antioxidant pathway [24]. Considering the dependency of *SDHB*-knockdown (*SDHB^KD^*) PCPG cells on antioxidant scavenging, in the present study, we aimed to determine the effectiveness of brusatol against *SDHB^KD^* PCPG.

## 2. Results

### 2.1. SDHB Deficiency Altered the Redox Balance in PCPG Cells

**Figure 1:**
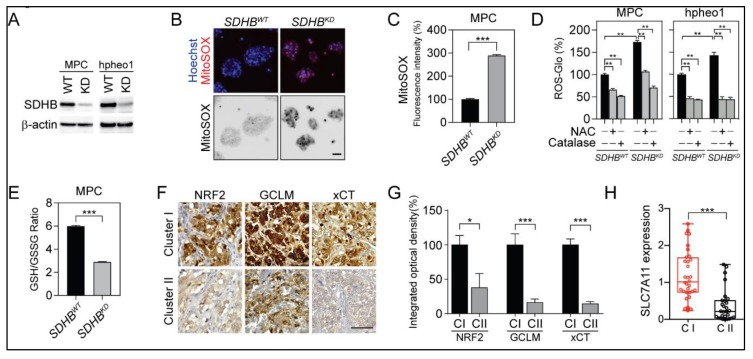
Succinate dehydrogenase subunit B (*SDHB*) deficiency reprogrammed reactive oxygen species (ROS) homeostasis and glutathione metabolism. (**A**) Immunoblotting showed the expression levels of *SDHB* in mouse pheochromocytoma (MPC) and hpheo1 cells. β-actin was used as internal control. (**B**) MitoSOX-Red staining showed ROS accumulation in *SDHB* knock down (*SDHB^KD^*) MPC cells. Bar = 10 μm. (**C**) Flow cytometry analysis showed elevated MitoSOX-Red staining in *SDHB^KD^* compared to *SDHB* wild type (*SDHB^WT^*) MPC cells. *** *p* < 0.001. (**D**) ROS quantification showed increased ROS in *SDHB^KD^* MPC and hpheo1 cells. Exogenous ROS scavenger *N*-acetylcysteine (NAC) and catalase reduced ROS level. ROS signal was measured and normalized to protein quantification. ** *p* < 0.01. (**E**) Glutathione quantification showed reduction of glutathione/glutathione disulfide (GSH/GSSG) ratio in *SDHB^KD^* compared to *SDHB^WT^* MPC cells. *** *p* < 0.001. (**F**) Immunohistochemistry staining showed that nuclear factor erythroid 2-related factor 2 (NRF2), glutamate-cysteine ligase regulatory subunit (GCLM), and cystine/glutamate transporter (*SLC7A11,* xCT) were increased in cluster I pheochromocytomas and paragangliomas (PCPGs). Bar = 50 μm. (**G**) Integrated optical density quantification for results shown in Figure 1F. For cluster I (CI), *n* = 4; for cluster II (CII), *n* = 4. * *p* < 0.05; *** *p* < 0.001. (**H**) Quantitative real-time PCR showed that SLC7A11 messenger RNA (mRNA) was increased in cluster I (C1; *n* = 8) compared to cluster II (CII; *n* = 7) PCPG specimen. *** *p* < 0.001.

Cancer-associated *SDHB* mutations have been found to result in the functional disruption of mitochondrial complex II, which causes catastrophic changes to cellular metabolism and redox homeostasis [25,26,27,28]. To better understand alterations in the redox status within *SDHB*-mutated PCPGs, we established *SDHB^KD^* PCPG cell lines based on the mouse pheochromocytoma cell line MPC (MPC *SDHB^KD^*) and the human cell line hpheo1 (hpheo1 *SDHB^KD^*). The knockdown efficiency was confirmed by western blot assay (Figure 1A). Further, the loss of *SDHB* led to substantial compromised oxidative metabolism and accumulation of succinate (Figure S1). Genetic disruption of *SDHB* resulted in robust accumulation of ROS in mitochondria and cytoplasm. MitoSOX Red staining showed a significant increase in ROS generation in *SDHB^KD^* compared to *SDHB* wild type (*SDHB^WT^*) cells (Figure 1B,C). A similar trend was observed through the direct quantification of intracellular H_2_O_2_, whereas the addition of the exogenous ROS scavengers *N*-acetylcysteine (NAC) or catalase reduced ROS accumulation (Figure 1D). The ROS elevation was relieved by the re-expression of *SDHB* (Figure S2A,B). To better understand the molecular basis of the redox balance in *SDHB*-deficient cells, we specifically quantified intracellular glutathione, the major source of ROS detoxification. The glutathione/glutathione disulfide (GSH/GSSG) ratio decreased by over 50% upon genetic silencing of *SDHB*, indicating that *SDHB^KD^* cells consume GSH for ROS scavenging and convert to more GSSG (Figure 1E and Figure S1C). Consistent with higher demands for glutathione, the expression levels of key enzymes, transcriptional factors, and transporters in the glutathione synthesis pathway, such as *NFE2L2*, glutamate-cysteine ligase regulatory subunit (*GCLM*), and cystine/glutamate transporter (*SLC7A11*), were upregulated in patients with cluster I PCPG (Figure 1F,G). Real-time PCR confirmed that the messenger RNA (mRNA) level of *SLC7A11* was increased in cluster I PCPGs (Figure 1H).

### 2.2. NRF2 Supported Glutathione De Novo Synthesis in SDHB^KD^ Cells

**Figure 2:**
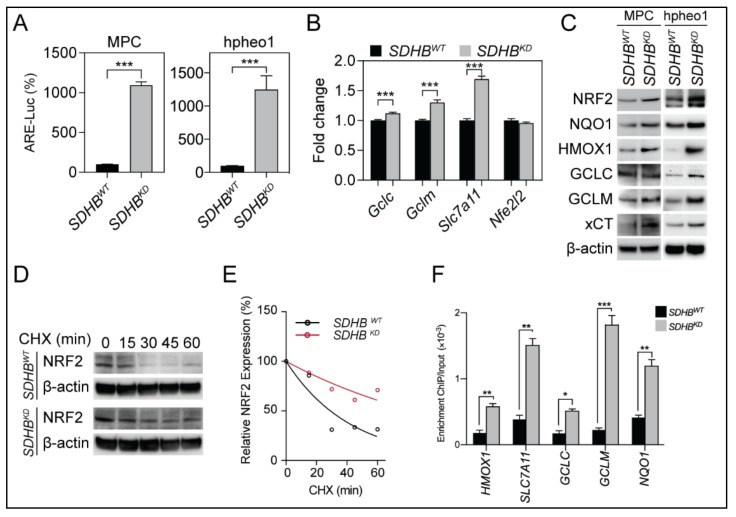
*SDHB* deficiency activated NRF2-driven glutathione synthetic pathway. (**A**) antioxidative response element (ARE)-luciferase reporter assay showed increased NRF2 activity in *SDHB^KD^* MPC and hpheo1 cells. *** *p* < 0.001. (**B**) Quantitative real-time PCR showed increased gene transcription of NRF2-associated genes *Gclm* and *Slc7a11* in *SDHB^KD^* MPC cells. *** *p* < 0.001. (**C**) Immunoblotting showed increased expression of NRF2 and its downstream targets in *SDHB^KD^* MPC and hpheo1 cells. β-actin was used as internal control. (**D**) Cycloheximide (CHX) pulse chase assay showed elevated NRF2 protein stability in *SDHB^KD^* hpheo1 cells. β-actin was used as internal control. (**E**) Quantification of NRF2 half-life from Figure 2D. (**F**) Chromatin immunoprecipitation (ChIP) PCR assay showed increased promoter affinity of NRF2 in *SDHB^KD^* hpheo1 cells. * *p* < 0.05; ** *p* < 0.01; *** *p* < 0.001.

The transcription of glutathione synthesis enzymes was regulated by NRF2, and increased levels of glutathione synthesis enzymes suggested functional alterations in NRF2 protein biology and transcriptional activity. To further understand the role of NRF2 in an *SDHB*-deficient genetic background, we analyzed the activation of NRF2 using an antioxidative response element (ARE) luciferase reporter assay. We found that *SDHB^KD^* cells exhibited significantly higher ARE transcriptional activity than *SDHB^WT^* cells, suggesting that the transcriptional activity of NRF2 was increased due to *SDHB* deficiency (Figure 2A). Introduction of *SDHB* expression decreased ARE luciferase activity in *SDHB^KD^* cells (Figure S2C). Quantitative real-time PCR and immunoblotting confirmed that NRF2 and its downstream target genes, such as *GCLM*, and *SLC7A11* (xCT), were significantly upregulated (Figure 2B,C). In addition, we found that the half-life of NRF2 was prolonged in *SDHB^KD^* cells compared to their wild-type counterparts, indicating a more sustained NRF2 activation (Figure 2D,E). Furthermore, a chromatin immunoprecipitation (ChIP) assay showed an increased affinity of NRF2 to the promoters of NAD(P)H dehydrogenase [quinone] 1 (*NQO1*), heme oxygenase (decycling) 1 (*HMOX1*), glutamate-cysteine ligase catalytic subunit (*GCLC*), *GCLM*, and *SLC7A11* in *SDHB^KD^* cells, confirming increased NRF2-mediated antioxidant gene transcription (Figure 2F).

### 2.3. Glutathione Synthesis Protected SDHB^KD^ Cells

**Figure 3:**
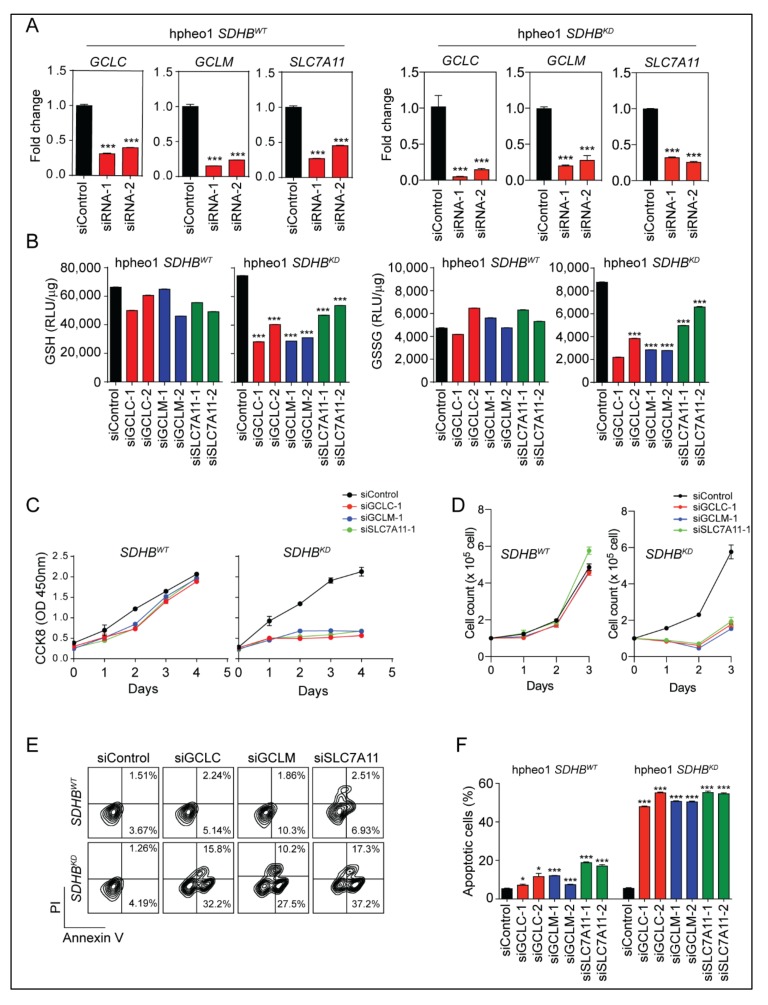
Glutathione synthesis is crucial for the survival and proliferation of *SDHB^KD^* cells. (**A**) Quantitative real-time PCR showed the knockdown efficiency of small interference ribonucleic acid (siRNA) targeting *GCLC*, *GCLM*, and *SLC7A11* in hpheo1 cells. *** *p* < 0.001. (**B**) Glutathione quantification assay showed that the cellular GSH and GSSG level was decreased in *SDHB^KD^* hpheo1 cells with siRNA targeting *GCLC*, *GCLM*, and *SLC7A11.* *** *p* < 0.001. (**C**) CCK8 assay showed that the cell viability of *SDHB^KD^* hpheo1 cells was suppressed with siRNAs targeting *GCLC*, *GCLM*, or *SLC7A11*. (**D**) Direct cell count showed reduced cell number of *SDHB^KD^* hpheo1 cells with siRNAs targeting *GCLC*, *GCLM*, or *SLC7A11*. (**E**) Annexin V/PI apoptosis assay showed the apoptotic changes of *SDHB^KD^* hpheo1 cells with siRNAs targeting *GCLC*, *GCLM*, and *SLC7A11*. (**F**) Quantification of apoptosis assay, *SDHB^KD^* hpheo1 cells showed increased cell apoptosis. * *p* < 0.05; *** *p* < 0.001.

The substantial upregulation of glutathione synthesis enzymes indicated the reprogramming of glutathione metabolism in *SDHB*-deficient cells, which might be essential to maintain cell survival under intense oxidative stress. To evaluate the role of the glutathione synthesis pathway, we designed and investigated small interfering RNA targeting *GCLC*, *GCLM*, and *SLC7A11* (Figure 3A and Figure S3A). The quantification of intracellular glutathione showed that the genetic silencing of glutathione synthesis enzymes depleted GSH levels in *SDHB^KD^* cells, whereas this effect was not seen in *SDHB^WT^* cells (Figure 3B). In addition, we evaluated the role of the glutathione synthesis pathway in cellular proliferation. The Cell Counting Kit-8 (CCK8) assay and direct cell counting showed that suppressing the glutathione synthesis pathway halted cellular proliferation in *SDHB^KD^* cells (Figure 3C,D and Figure S3B). Moreover, annexin V/propidium iodide (PI) flow cytometry revealed potent apoptotic changes in *SDHB^KD^* cells when the glutathione synthesis pathway was suppressed, whereas this phenomenon was not seen in *SDHB^WT^* cells (Figure 3E,F and Figure S3C,D). These data demonstrated that *SDHB* deficiency established a dependency on enhanced glutathione synthesis, which not only relieved the oxidative stress but also supported cellular physiology in oncogenic pathways. This finding also suggests that the glutathione antioxidant pathway could be a valuable therapeutic target for malignancies with *SDHB* deficiency.

### 2.4. The NRF2 Inhibitor Brusatol Disrupted Glutathione Synthesis

**Figure 4:**
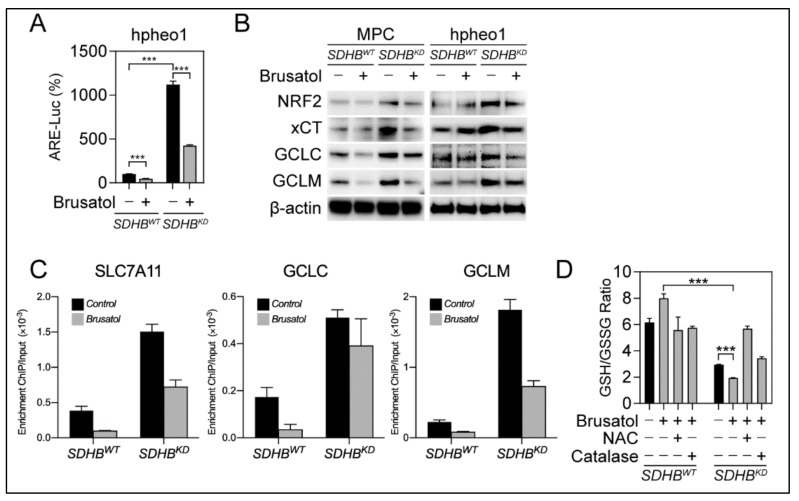
Suppressing of NRF2 activity inhibits glutathione synthesis pathway in *SDHB^KD^* cells. (**A**) ARE-luciferase reporter assay showed brusatol treatment inhibited NRF2 transcriptional activity in *SDHB^KD^* hpheo1 cells. *** *p* < 0.001. (**B**) Immunoblotting showed that brusatol suppressed the expression of NRF2, xCT, GCLC, and GCLM in MPC and hpheo1 cells. β-actin was used as internal control. (**C**) ChIP PCR assay showed that Brusatol reduced promoter affinity of NRF2 to SLC7A11, GCLC, and GCLM. (**D**) Glutathione quantification showed that brusatol reduced GSH/GSSG ratio in *SDHB^KD^* MPC cells. Exogenous ROS scavengers NAC and Catalase can restore the ratio with the presence of Brusatol. *** *p* < 0.001.

We investigated whether brusatol could be effective for *SDHB^KD^* PCPG cells due to their dependency on antioxidant scavenging. The ARE-luciferase reporter assay showed that brusatol potently eliminated NRF2-derived transcriptional activity in *SDHB^KD^* cells (Figure 4A). This finding was confirmed by immunoblotting, evidenced by a reduction in the expression of NRF2, GCLC, GCLM, and xCT upon brusatol treatment (Figure 4B). Accordingly, ChIP PCR showed that the affinity of NRF2 to the promoters of *GCLC*, *GCLM*, and *SLC7A11* was impaired after brusatol treatment (Figure 4C). In addition, we found that brusatol reduced the half-life of NRF2 protein and prompted NRF2 ubiquitination (Figure S4A–C). The suppression of the glutathione synthesis pathway further reduced the availability of GSH for ROS scavenging, as the GSH/GSSG ratio decreased after brusatol treatment in *SDHB^KD^* cells, whereas the ROS scavengers NAC and catalase could restore GSH levels (Figure 4D and Figure S4D). These results indicate that the glutathione synthesis pathway is one of the primary targets within brusatol-induced NRF2 inhibition.

### 2.5. NRF2 Suppression Led to Oxidation-Derived Cellular Damage

**Figure 5:**
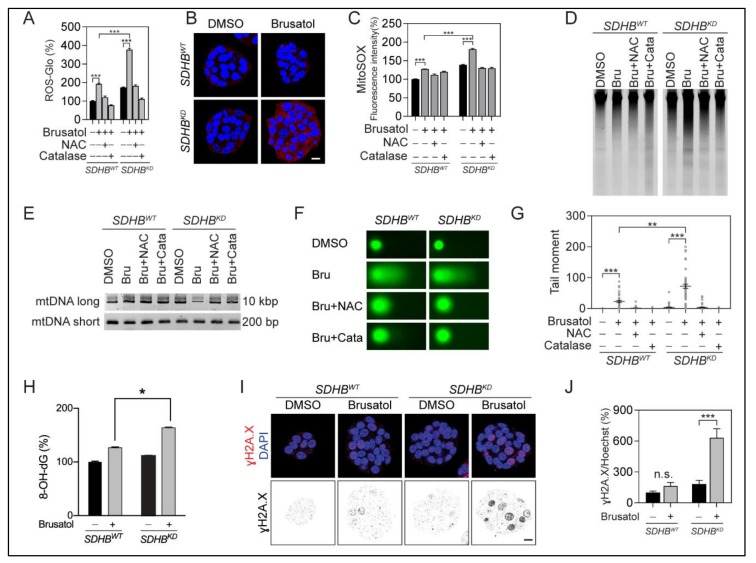
Brusatol disrupted ROS homeostasis and led to oxidative DNA damage. (**A**) ROS quantification assay showed that brusatol increased ROS level, especially in *SDHB^KD^* MPC cells. Exogenous ROS scavengers restored ROS to the normal level. ROS signal was measured and normalized to protein quantification. *** *p* < 0.001. (**B**) MitoSOX-Red staining showed increased ROS level in *SDHB^KD^* MPC cells after brusatol treatment. Bar = 10 μm. (**C**) Flowcytometry analysis showed increased MitoSOX-Red signal in *SDHB^KD^* cells after brusatol treatment. ROS scavengers restored ROS to normal level. Dimethyl sulfoxide (DMSO) was used as solvent control. Cell nuclei were labeled with 4′,6-diamidino-2-phenylindole (DAPI). Bar = 10 μm. (**D**) Total genomic DNA electrophoresis showed increased DNA fragmentation in *SDHB^KD^* MPC cells with brusatol treatment. (**E**) Mitochondrial deoxyribonucleic acid (mtDNA) PCR assay showed the long and short fragments from the mitochondrial genome of MPC cells was performed after brusatol treatment. Exogenous ROS scavengers restored DNA fragmentation. (**F**) Comet assay showed increased DNA fragmentation (Comet tail) in *SDHB^KD^* MPC cells. Exogenous ROS scavengers restored DNA fragmentation. (**G**) Quantification of comet assay in Figure 5F. ** *p* < 0.01; *** *p* < 0.001. (**H**) Oxidative DNA damage ELISA assay showed stronger elevation of 8-OH-dG in *SDHB^KD^* MPC cells with brusatol treatment. * *p* < 0.05. (**I**) Immunostaining showed elevated γH2A.X in *SDHB^KD^* MPC cells with brusatol treatment. Bar = 10 μm. (**J**) Quantification of γH2A.X in Figure 5I. *** *p* < 0.001.

The suppression of the NRF2 antioxidative pathway depleted the intracellular pool of GSH, which may translate into oxidation-derived cellular damage. Both the ROS-Glo and MitoSOX Red staining assays showed that the ROS level remarkably increased after brusatol treatment (Figure 5A–C). High ROS levels in *SDHB^KD^* cells resulted in oxidative damage in both mitochondrial and genomic DNA (Figure 5D,E and Figure S6G,H). The comet assay confirmed increased DNA fragmentation in *SDHB^KD^* cells treated with brusatol, whereas exogenous ROS scavengers salvaged DNA fragmentation in the same assay (Figure 5F,G). Furthermore, DNA oxidative damage ELISA and γH2A.X staining confirmed oxidative DNA damage in *SDHB^KD^* cells with brusatol (Figure 5H–J).

### 2.6. Selective Vulnerability of SDHB^KD^ Cells to NRF2 Suppression

**Figure 6:**
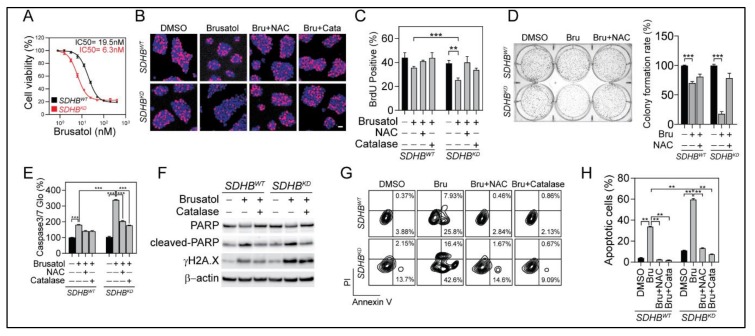
Brusatol selectively suppressed *SDHB^KD^* cells. (**A**) Dose-response curve showed that *SDHB^KD^* MPC cells were more vulnerable to Brusatol treatment. (**B**) BrdU (red) incorporation assay shows that Brusatol selectively inhibited the proliferation of *SDHB^KD^* MPC cells. Cell nuclei were labeled with Hoechst 33342 (blue). Bar = 20 μm. (**C**) Quantification of BrdU-positive cells in Figure 6B. ** *p* < 0.01; *** *p* < 0.001. (**D**) Long-term colony formation assay and quantification showed that Brusatol inhibited cluster formation of *SDHB^KD^* MPC cells. *** *p* < 0.001. (**E**) Caspase 3/7-Glo assay showed significantly increased Caspase 3/7 activity in *SDHB^KD^* MPC cells with Brusatol. Luminescence was measured and normalized to protein quantification. *** *p* < 0.001. (**F**) Immunoblotting showed increased cleaved-poly (ADP-ribose) polymerase (PARP) and γH2A.X after Brusatol treatment. β-actin was used as internal control. (**G**) Annexin V/PI apoptosis analysis showed that *SDHB^KD^* MPC cells exhibited more apoptotic cells under Brusatol treatment compared with *SDHB^WT^* cells. ROS scavengers reduced the number of apoptotic cells. (**H**) Quantification of apoptotic cells from Figure 6G. ** *p* < 0.01.

As a consequence of elevated oxidative stress, we recorded profound and selective cytotoxicity in *SDHB^KD^* cells upon brusatol treatment. A cell viability assay showed *SDHB^KD^* cells to be more vulnerable to brusatol treatment with an IC_50_ of 6.3 nM for *SDHB^KD^* cells compared with an IC_50_ of 19.5 nM for *SDHB^WT^* cells (Figure 6A). A 5-bromo-2’-deoxyuridine (BrdU) incorporation assay confirmed that brusatol reduced cell proliferation by 35.3% in *SDHB^KD^* cells, whereas an 18.8% reduction was observed in the wild-type counterpart (Figure 6B,C). Similarly, a long-term colony-formation assay revealed that brusatol significantly reduced the colony count in *SDHB^KD^* but not *SDHB^WT^* cells (Figure 6D). Importantly, the brusatol-mediated reduction in cell proliferation could be rescued by the ROS scavengers NAC or catalase, suggesting the involvement of an overload of oxidative damage. Furthermore, a caspase 3/7 activity assay showed higher apoptotic changes in *SDHB*-deficient cells (Figure 6E). An immunoblotting assay confirmed caspase cleavage, accompanied with PARP cleavage and γH2A.X elevation in brusatol-treated *SDHB^KD^* cells (Figure 6F). In agreement with these findings, annexin V/PI flow cytometry showed an increased number of apoptotic cells in *SDHB^KD^* cells compared with *SDHB^WT^* after receiving the same dosage of brusatol (Figure 6G,H). The combination with the ROS scavengers NAC and catalase significantly reduced brusatol-induced cytotoxicity, indicating that ROS imbalance is the primary cause of growth arrest and apoptotic changes.

### 2.7. NRF2 Blockade for SDHB-Mutated PCPGs

**Figure 7:**
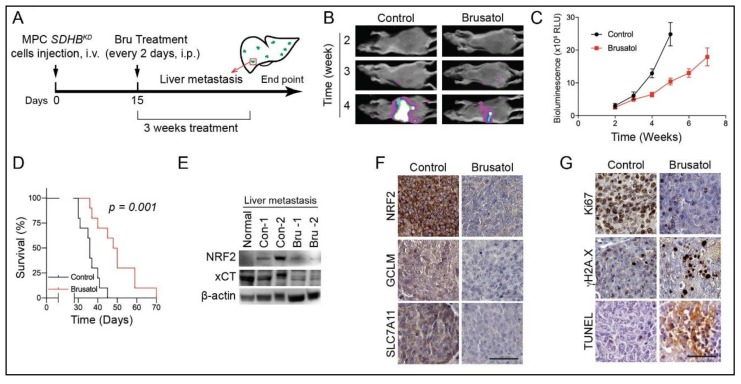
Brusatol suppressed *SDHB*-mutated MPC allograft in vivo. (**A**) Schematic illustration for the allograft and treatment schedule. (**B**) Luciferase imaging showed that brusatol treatment suppressed *SDHB^KD^* hepatic lesions in vivo. (**C**) Quantification of tumor volume shown in Figure 7B. (**D**) Kaplan–Meier analysis showed that brusatol prolonged overall survival of tumor-bearing animal (*p* = 0.001). (**E**) Immunoblotting showed that Brusatol inhibited the expression of NRF2 and xCT hepatic metastatic lesions. β-actin was used as internal control. (**F**) Immunohistochemistry assay showed that the expression of NRF2, xCT, and GCLM were suppressed in metastatic lesions under Brusatol treatment. Bar = 50 μm. (**G**) Immunohistochemistry assay showed the staining of Ki67, γH2A.X, and TUNEL assay in tumor sections. Bar = 50μm.

Considering the central roles in maintaining ROS homeostasis for *SDHB^KD^* cells, targeting the NRF2 antioxidant pathway could be a valuable therapeutic strategy as a selective treatment for *SDHB*-deficient malignancies. To test this hypothesis, we investigated the tumor-suppressing effect of brusatol in a preclinical animal model bearing metastatic PCPG allografts. We established a liver metastasis model by tail vein injection of MPC *SDHB^KD^* cells in NU/J mice. Mice were randomized and administered 1 mg kg^−1^ brusatol intraperitoneal (i.p.) every other day (Figure 7A). In vivo luminescence imaging showed that brusatol significantly reduced the speed of tumor metastasis (Figure 7B,C). Moreover, brusatol improved disease outcome with significantly prolonged overall survival by 36.1% (36 days versus 49 days, Figure 7D). Furthermore, the immunoblotting assay showed that brusatol suppressed the expression of NRF2 and xCT in the metastatic lesions (Figure 7E). Consistent with this, immunohistochemistry showed that the expression levels of NRF2, as well as the glutathione metabolic enzymes GCLM and xCT, were diminished in the brusatol-treated group (Figure 7F). In addition, low expression of the proliferation marker Ki67 but higher expression of the DNA damage marker γH2A.X/Terminal deoxynucleotidyl transferase dUTP nick end labeling (TUNEL) were observed in brusatol-treated tissue specimens (Figure 7G). However, for animals bearing MPC *SDHB^WT^* liver metastasis, brusatol did not effectively suppressed tumor growth, as on the *SDHB*-deficient cells (Figure S5A,B). The median survival day was slightly extended from 38.5 days to 43 days (*p* = 0.3424; Figure S5C). Taken together, these findings demonstrated that *SDHB*-deficient PCPG cells are sensitive to oxidative stress induced by suppressing NRF2 activity.

## 3. Discussion

In the present study, we demonstrated that cluster I PCPG with *SDHB* deficiency exhibited a unique metabolic pattern, characterized by a high ROS burden and dependency on de novo glutathione synthesis. We showed that *SDHB^KD^* cells developed a dependency on NRF2 anti-oxidative pathways to meet the increased needs of ROS detoxification to avoid cell death. NRF2 stabilization and transactivation supported de novo glutathione synthesis through the upregulation of GCLC, GCLM, and SLC7A11. Targeting the NRF2/glutathione axis resulted in ROS overload, oxidative DNA damage, reduced cellular proliferation, and profound cytotoxicity. Moreover, brusatol, a potent NRF2 inhibitor, established synthetic lethality with *SDHB* deficiency, which significantly improved disease outcome with prolonged overall survival in vivo. Our findings highlight the important role of the NRF2/glutathione axis in cluster I PCPG with *SDHB* deficiency and a potential therapeutic approach for these and other *SDHB*-related tumors.

*SDHB* is the iron-sulfur subunit of mitochondrial complex II, which plays a critical role in succinate dehydrogenase activity. Pathogenic mutations in *SDHB* result in abnormalities in electron transfer steps within mitochondrial complex II, which compromise the citric acid cycle and establish a pseudohypoxia phenotype, Warburg-like metabolism, and the formation of several types of human malignancies [14,29,30]. Moreover, the loss of the SDHB subunit prompts the accumulation of electrons in the flavin group in SDHA, which promotes superoxide generation through the autoxidation of the reduced flavin group by O_2_ in the matrix [25]. In the present study, we confirmed that the accumulation of cellular ROS is closely related to the dysfunction of SDHB (Figure 1A–D). The glutathione synthesis pathway was activated as a consequence of elevated intracellular ROS (Figure 1E–H). NRF2, the master transcriptional factor for redox regulation, was stabilized and initiated the transcription of antioxidant genes such as *NQO1*, *HMOX1*, *GLCL*, *GCLM*, and *SLC7A11* (Figure 2). Importantly, we discovered that *SDHB*-mutated cells exhibited a dependency on glutathione consumption, as genetic silencing of glutathione synthesis enzymes compromised cellular viability specifically in cells with SDHB reduction (Figure 3). These findings highlight a metabolic signature in *SDHB*-mutated PCPG, with markedly enhanced ROS production and scavenging.

Owing to highly harmful oxidative-derived cellular damage, the intracellular ROS level is strictly controlled to a minimal level to protect macromolecules such as DNA, proteins, and lipids. Several evolutionarily conserved mechanisms have been identified to eliminate ROS from cellular compartments. For example, superoxide dismutases (SODs) are enzymes that catalyze the superoxide radical (O_2_^−^) into oxygen (O_2_) or hydrogen peroxide (H_2_O_2_). Glutathione peroxidases (GPXs) are a family of enzymes that transform H_2_O_2_ into water in a glutathione/reduced form nicotinamide adenine dinucleotide phosphate (NADPH)-dependent manner [31,32]. The transcription level of these antioxidant genes is regulated by NRF2, which governs the production and scavenging of intracellular ROS [33,34]. ROS accumulation in the cytoplasm compromises the function of kelch-like ECH-associated protein 1 (KEAP1), the E3 ligase that deactivates NRF2, leading to the stabilization and functioning of NRF2 to activate the antioxidant pathway [23,34,35]. In the present study, we confirmed that in cells with *SDHB* deficiency, the high ROS levels are sufficient to stabilize NRF2 (Figure 2D,E). NRF2 translocates into the nucleus and mediates the transcriptional activation of several key antioxidant genes such as *GCLC*, *GCLM*, and *SLC7A11* (Figure 2F). The enhancement of the glutathione synthesis pathway was confirmed in patient-derived specimens, as the enzymes were upregulated in *SDHB*-defective cluster I PCPG (Figure 1F–H). These findings highlight that the NRF2-derived glutathione synthesis pathway plays a critical role in *SDHB*-deficient malignancies, which is a key clue to the therapeutic vulnerability for this disease cluster.

In the present study, the NRF2-derived de novo glutathione synthesis pathway was targeted as a therapeutic approach for PCPGs with *SDHB* deficiency. Brusatol has been repeatedly shown to be a potent inhibitor for NRF2 and antioxidant pathways [24,36,37,38,39]. Several studies have indicated that brusatol alone exhibits mild cytotoxic effects and is frequently evaluated as a sensitization approach to support other cytotoxic therapies such as radiation therapy and cisplatin [24,40,41]. Interestingly, our in vitro assays and preclinical animal models showed that brusatol effectively suppressed *SDHB*-mutated malignancies as a single therapeutic agent (Figure 5 and Figure 6). We believe that in malignancies with intrinsic metabolic deficiencies, such as *SDHB*-mutated PCPG, elevated cytosolic ROS synergizes with antioxidant pathway inhibition. The synthetic lethality approach is sufficient in causing overwhelming oxidative-derived damage in mitochondrial and genomic DNA, which proceeds to apoptotic changes and cancer suppression.

## 4. Materials and Methods

### 4.1. Study Design

This study was designed to evaluate the role of NRF2-govern glutathione synthesis axis in *SDHB*-deficient PCPG and explore potential therapeutic targets. In this study, we tested our hypothesis on the basis of two previously reported PCPG models: mouse pheochromocytoma (MPC) and human pheochromocytoma (hpheo1) cells, through both in vitro and in vivo assays. Clinical samples from patients with cluster I and II PCPG were used as validation. All experiments were replicated three times, and the key experiments were repeated in two different cell line models. The investigators were not blinded, and data were collected and analyzed objectively.

### 4.2. Patient Samples

Tumor samples from cluster I and cluster II PCPG patients were dissected from the clinical specimens. This study was approved by the Institutional Review Board of the *Eunice Kennedy Shriver* National Institute of Child Health and Human Development (NICHD), National Institutes of Health (NIH), and all patients gave written informed consent. The IRB Protocol #: 00CH0093.

### 4.3. Cell Lines

Mouse pheochromocytoma (MPC) cell lines were cultured in Dulbecco’s modified Eagle’s medium (DMEM) containing 10% fetal bovine serum (FBS), penicillin, and streptomycin (Thermo Fisher, Waltham, MA, USA). *SDHB* wild type (*SDHB^WT^*) and *SDHB* knock down (*SDHB^KD^*) MPC cells were described previously [20]. Progenitor cell line derived from human pheochromocytoma (hpheo1) was a gift from Dr. Hans Ghayee approved under material transfer agreement, Eunice Kennedy Shriver NICHD [42]. *SDHB*-deficient hpheo1 was prepared by lentivirus with short hairpin RNA targeting *SDHB* (V3SVHS00_8112304, targeting sequence CAG AGC TGA ACA TAA TTT A, GE Dharmacon, Boston, MA, USA). Puromycin selection was performed to establish isogenic cells, and knockdown efficiency was confirmed by immunoblotting.

### 4.4. Reagents and Treatment Condition

Brusatol, *N*-acetylcysteine (NAC) and catalase were purchased from Sigma (St. Louis, MO, USA) and dissolved in phosphate-buffered saline (PBS). The final concentration used in the present study was Brusatol 40 nM, NAC 2.5 mM, and catalase 500 U mL^−1^. For gene expression analysis and oxidative stress and DNA damage-related experiments, cells were treated with indicated conditions for 24 h. For cytotoxicity related experiments, cells were under treatment for 72 h.

### 4.5. Real-Time PCR

Total RNA was extracted from MPC and hpheo1 cells using PureLink RNA mini kit (Thermo Fisher), and reverse transcript to DNA using Superscript IV VILO Master Mix (Thermo Fisher). Real-time PCR was performed using Power SYBR Green Master Mix. Primers used in the current study included: mGCLC.F: 5′-CTA CCA CGC AGT CAA GGA CC-3′; mGCLC.R: 5′-CCT CCA TTC AGT AAC AAC TGG AC-3′; mGCLM.F: 5′-ACT CAC AAT GAC CCG AAA GAA C-3′; mGCLM.R: 5′-CCT GCT CTT CAC GAT GAC CG-3′; mSLC7A11.F: 5′-GGC ACC GTC ATC GGA TCA G-3′; mSLC7A11.R: 5′-CTC CAC AGG CAG ACC AGA AAA-3′; hNQO1 (QT00050281); hHMOX1 (QT00092645); hNFE2L2 (QT00027384); hSLC7A11 (QT00002674); and hACTB (QT00095431).

### 4.6. RNA Interference

Small interference RNA (siRNA) oligos were designed and synthesized by Integrated DNA Technologies (IDT, Coralville, IA, USA). The sequences of siRNA used in the study are listed as follows: siGCLC.1.F: 5′-ACA AUU GGA CAG AUA GUA GCC AAC UGA-3′; siGCLC.1.R: 5′-AGU UGG CUA CUA UCU GUC CAA UUG T-3′; siGCLC.2.F: 5′-UAA AUA UUG GUA CAU UGA UGA CAA CCU-3′; siGCLC.2.R: 5′-GUU GUC AUC AAU GUA CCA AUA UUT A-3′; siGCLM.1.F: 5′-AAG GUU UUU UGG AUA CAA UCA UGA AGC-3′; siGCLM.1R: 5′-UUC AUG AUU GUA UCC AAA AAA CCT T-3′; siGCLM.2.F: 5′-CCU UCU UUU AGC UUG UAA AAU GUA GCC-3′; siGCLM.2.R: 5′-CUA CAU UUU ACA AGC UAA AAG AAG G-3′; siSLC7A11.1.F: 5′-AUG ACU GUG CUU CCA AGU AUG CAU CUA-3′; siSLC7A11.1.R: 5′-GAU GCA UAC UUG GAA GCA CAG UCA T-3′; siSLC7A11.2.F: 5′-UUC UUU AUA GUU GUU CCC AAU UCA GCA-3′; siSLC7A11.2.R: 5′-CUG AAU UGG GAA CAA CUA UAA AGA A-3′. AllStar negative control siRNA (QIAGEN, Hilden, Germany) was used as a control.

### 4.7. Chromatin Immunoprecipitation (ChIP) Assay

The ChIP assay was performed by using a ChIP-IT High Sensitivity kit (Active Motif, Carlsbad, CA, USA) according to the manufacturer’s protocol. A total of 15 million cells were fixed and collected for nuclei isolation. Chromatin was prepared from isolated nuclei, sheared by sonication, and precipitated with anti-NRF2 antibody (Active Motif). Promoter enrichment was quantified by quantitative real-time PCR assay comparing pull-down DNA and input DNA. The sequences of the primer set used are listed as follows: HMOX1.F: 5′-ACA AAG GGA AGG CGG ATT TT-3′; HMOX1.R: 5′-ACT TCC TCC TGC CTA CCA TT-3′; SLC7A11.F: 5′-AGC TTC CCA CAA AGT CGA AG-3′; SLC7A11.R: 5′-ACA TTC CTG CTT GTC TTG GT-3′; GCLC.F: 5′-CGC AGT TGT TGT GAT ACA GCC-3′; GCLC.R: 5′-GGA CTG AGA CTT TGC CCT AAG AA-3′; GCLM.F: 5′-ATT CCA AAC TGA GGG AGC TGT TT-3′; 5′-GCLM.R: ATG AGT AAC GGT TAC GAA GCA CT-3′; NQO1.F: 5′-GTG TGA CAG AGG CCT CAA AA-3′; NQO1.R: 5′-TGA TCC CTG GAC TCT CTT GG-3′.

### 4.8. ROS Quantification

Cellular ROS was measured using ROS-Glo H_2_O_2_ assay kit (Promega, Madison, WI, USA) according to the manufacturer’s protocol. The luminescence signal was measured using Polarstar Optima plate reader (BMG LABTECH, Ortenberg, Germany). Mitochondrial ROS were measured using MitoSOX staining (Thermo Fisher). Cells were incubated with 5 μM MitoSOX-Red at 37 °C for 10 min and analyzed by either confocal imaging using Zeiss 710 NLO or flow cytometry using LSRFortessa SORP or FACSCanto II. Fluorescence intensity was measured and quantified by using ImageJ software (v1.8.0_112).

### 4.9. ARE Luciferase Reporter Assay

ARE transcriptional activity was determined using either transfection of reporter plasmid pGL4.37-luc2P-ARE-Hygro (Promega, Madison, WI, USA) or Cignal Antioxidant Response Reporter system (Qiagen) according to the manufacturer’s protocol. For dual luciferase assay, 900 ng pGL3.47-ARE-Luc and 100 ng pRL-TK (Promega) were transfected into 10,000 cells using Lipofectamine 2000 (Thermal Fisher, Waltham, MA, USA). Luminescence signal was recorded using Polarstar Optima plate reader and normalized to protein quantification or Renilla luciferase activity.

### 4.10. Immunoblotting

Total protein was extracted from cultured cells using RIPA lysis buffer supplemented with protease and phosphatase inhibitor cocktail (Thermo Fisher). The cell lysates were resolved on Bis-Tris gel (Thermo Fisher) and transferred to polyvinylidene difluoride (PVDF) membrane (Millipore, Burlington, MA, USA). After blocking in Superblock (Thermo Fisher), the membrane was incubated with primary antibodies at 4 °C overnight. Protein was further probed by horseradish peroxidase (HRP)-conjugated secondary antibodies and visualized using a Bio-Rad ChemiDoc Imaging System. The antibodies used in the present study included: NRF2 (CST, Danvers, MA, 1:1000), GCLC (Abcam, Cambridge, United Kingdom, 1:1000), GCLM (Proteintech, Rosemont, IL, 1:1000), xCT (Abcam, 1:1000), NQO1 (Abcam, 1:1000), Ubiquitin (Abcam, 1:2000), PARP1 (CST, 1:1000), γH2A.X (CST, 1:1000), EGFP (Thermo Fisher, 1:1000), Human influenza hemagglutinin (HA)-tag (Covance, Princeton, NJ, 1:1000), DYKDDDDK peptide tag (DDK, Origene, Rockville, MD, 1:2000), and β-actin (Sigma, 1:5000). All immunoblotting images are shown in Figure S6.

### 4.11. Immunofluorescence Staining

Cells were seeded in 8-well chamber slides (Ibidi, Martinsried, Germany), fixed in 4% paraformaldehyde and permeabilized with 0.3% TritonX-100. Cells were blocked in Superblock (Thermo Fisher) and incubated with γH2A.X antibody (CST, 1:200) at 4 °C overnight. Cells were washed three times with PBS and incubated with fluorescent conjugated secondary antibody (Thermo Fisher). Images were taken using a Zeiss LSM710 confocal microscope (Oberkochen, Germany).

### 4.12. Annexin V/PI Apoptosis Assay

Cellular apoptosis was analyzed by Annexin V/PI apoptosis kit (Thermo Fisher) according to the manufacturer’s manual. Cells were lifted by trypsin and stained with annexin V-Alexa 488 and PI in binding buffer for 20 min on ice. Fluorescent signal was analyzed using a FACS Canto II flow cytometer (BD, Franklin Lakes, NJ, USA).

### 4.13. Caspase-3/7 Activity Assay

A total of 5000 cells were seeded in a 96-well plate and caspase 3/7 activity was measured using a Caspase-Glo 3/7 assay (Promega) according to the manufacturer’s manual. Luminescence signal was measured by a Polarstar Optima plate reader and normalized to protein quantification.

### 4.14. DNA Oxidative Damage ELISA Assay

DNA oxidative damage was measured using Cayman DNA/RNA Oxidative Damage ELISA kit (Cayman Chemical, Ann Arbor, MI, USA), according to the manufacturer’s protocol. Total DNA was extracted from cultured cells and digested using nuclease P1. A total of 100 ng of DNA was loaded into a microwell plate and DNA/RNA oxidative damage was measured by ELISA assay.

### 4.15. DNA Fragmentation Assay

Cells were harvested and total DNA was isolated and purified using QIAamp DNA Blood kit (QIAGEN). Then, 500 ng of DNA was resolved by electrophoresis using 4–20% Tris-borate-EDTA (TBE) gel (Thermo Fisher). Gel was stained with SYBR Safe (Thermo Fisher) and exposure using a Bio-Rad ChemiDoc Imaging System. Mitochondrial DNA damage was measured as previously described [43]. A long fragment (10.1 kb) of mitochondrial DNA (mtDNA) was amplified using primers mtDNA.long.F: 5′-GCC AGC CTG ACC CAT AGC CAT AAT AT-3′ and mtDNA.long.R: 5′-GAG AGA TTT TAT GGG TGT AAT GCG G-3′. A short fragment (241 bp) mtDNA was amplified with the primers mtDNA.short.F: 5′-CCT CCC ATT CAT TAT CGC CGC CCT TGC-3′ and mtDNA.short.R: 5′-GTC TGG GTC TCC TAG TAG GTC TGG GAA-3′.

### 4.16. Comet Assay

Alkaline Comet assay was performed to evaluate the DNA damage as we described before [44]. Cells were harvested, diluted in low temperature melting agarose, and spread on pre-coated glass slides. After lysis, the slides were subjected to electrophoresis (voltage: 1 V cm^−1^) for 1 h at room temperature in dark. Each slide was stained with SYBR Green and evaluated by confocal microscopy.

### 4.17. BrdU-Incorporated Cell Proliferation

BrdU cell proliferation assay was performed as previous described [20]. Generally, cells were incubated with 10 µM of BrdU for 2 h and fixed in 4% paraformaldehyde (PFA). Cells were then treated with 2N HCl and neutralized with 0.1M sodium borate. Cells were stained with mouse anti-BrdU antibody (0.5 µg per test, BD Biosciences) overnight at 4 °C. After staining with donkey anti-mouse immunoglobulin G (IgG) Alexa Fluor 488, fluorescent signal was recorded by confocal microscopy.

### 4.18. Colony Formation Assay

*SDHB^WT^* and *SDHB^KD^* MPC cells were seeded in a 6-well plate at a density of 1000 cells per well. Cells were treated with compounds for 2 weeks, fixed in 4% cold formaldehyde (Sigma), and stained with 2% crystal violet (Sigma). This experiment was repeated by three replicates, and the images were quantified by ImageJ.

### 4.19. Cell Viability Analysis

The cell counting kit-8 (CCK8, Dojindo, Rockville, MD, USA) was performed to determine cell viability. CCK8 was added at a ratio of 1:20 to cell media and incubated at 37 °C for 1 h. The absorbance of optical density (OD) 450 nm was measured by an Epoch plate reader (BioTek, Winooski, VT, USA). Each sample was done in triplicate and results are presented as mean ± standard error of mean (SEM).

### 4.20. Immunoprecipitation and Ubiquitination Assay

Immunoprecipitation was performed as previously described [45]. NRF2-myc-DDK and Ubiquitin-HA plasmids were transfected into hpheo1 *SDHB^WT^* and *SDHB^KD^* cells using lipofectamine 3000 (Thermo Fisher). Cells were harvested and lysed in ice-cold RIPA buffer supplemented with protease/phosphatase inhibitor cocktail (Thermo Fisher) and 1% SDS. NRF2 was pulled down by protein G Dynabeads (Thermo Fisher) and antibody against DDK. Bounded protein was eluted and analyzed by Western blot.

### 4.21. CHX Pulse Chase Protein Half-Life Measurement

CHX pulse chase assay was performed as previously described [46]. NRF2-EGFP plasmid was transfected into hpheo1 *SDHB^WT^* and *SDHB^KD^* cells. Cells were treated with brusatol overnight. Cells were exposed to 200 μg mL^−1^ CHX (Sigma). Samples were collected at different time points and analyzed by Western blot.

### 4.22. Metastatic Allograft Mouse Model

The animal experiments were conducted with the principles and procedures outlined in the Eunice Kennedy Shriver NICHD Guide for the Care and Use of Animals and approved by the Animal Care and Use Committee of the National Institute of Health (Animal Study Protocol: 15-028). MPC *SDHB^KD^* Luc cells (1.5 million) suspended in normal phosphate-buffered saline (PBS) solution were injected into the tail vein of female athymic mice (Ncr-nu/nu, aged 8 weeks). Ten days after inoculation, animals were screened by IVIS imaging system to evaluate the presence of metastatic lesions. Mice were randomized into two groups (10 mice per group) and treated with PBS or brusatol. Brusatol was injected intraperitoneal (i.p.) every other day at 1 mg kg^−1^. All animals were carefully monitored every day and tumor growth was recorded weekly by IVIS in vivo imaging system. At the end of the experiments, all animals were sacrificed, and liver metastatic lesions were harvested for further analysis [47].

### 4.23. Immunohistochemistry and TUNEL Assay

Immunohistochemistry was performed in tumor sections, as reported previously [20]. Formalin-fixed, paraffin-embedded (FFPE) tissue slides were incubated with NRF2 (Abcam, ab62352), xCT (Abcam, ab37185), GCLM (Proteintech, 14241-1-AP), and Ki67 (Abcam, ab15580) antibodies and followed with 3,39-diaminobenzidine (DAB) substrate. Slides were counter-stained by hematoxylin and visualized by light microscope.

### 4.24. Statistical Analysis

Statistical analysis was performed using Student’s *t*-Test between two groups. Differences among groups were analyzed using one-way ANOVA test followed by Student’s *t*-Test as the post-statistical analysis. All tests were two-sided, the results were presented as mean ± SEM. A *p*-value < 0.05 was considered as statistically significant. All of the analysis was conducted using GraphPad Prism 7.01 (GraphPad Software, San Diego, CA, USA).

## 5. Conclusions

In summary, we demonstrated that *SDHB*-deficient cluster I PCPG exhibited high ROS generation, leading to a dependency on NRF2-driven de novo glutathione synthesis. Targeting the anti-oxidative pathways of the master transcription factor NRF2 could be a new approach to treat this type of cancer through synthetic lethality with intrinsic ROS burden.

## Supplementary Materials

The following are available online at https://www.mdpi.com/2072-6694/12/2/280/s1, Figure S1: Metabolic characterization of *SDHB*-deficient cells, Figure S2: Ectopic expression of SDHB restored redox balance in SDHB-deficient cells, Figure S3: Glutathione synthesis is crucial for the survival in *SDHB*-deficient MPC cells, Figure S4: Brusatol induced NRF2 ubiquitination and degradation and suppressed glutathione synthesis, Figure S5: Brusatol treatment in SDHB wild-type MPC allograft in vivo, Figure S6: Western Blot and DNA electrophoresis images.
