# Supplementary material for: Targeting NRF2-Governed Glutathione Synthesis for SDHB-Mutated Pheochromocytoma and Paraganglioma
Source: Cancers (Basel). 2020 Jan 23;12(2):280. doi: 10.3390/cancers12020280 (PMC7072390; doi:10.3390/cancers12020280)
Supplement: Supplementary file 1 [file cancers-12-00280-s001.pdf]

Supplementary Materials

Targeting NRF2-Governed Glutathione Synthesis for *SDHB*-Mutated Pheochromocytoma and Paraganglioma

Yang Liu, Ying Pang, Veronika Caisova, Jianyi Ding, Di Yu, Yiqiang Zhou, Thanh-Truc Huynh, Hans Ghayee, Karel Pacak and Chunzhang Yang

1. Materials and Methods

1.1. Succinate Assay

Cellular level of succinate was determined by Succinate Assay Kit (Abcam) according to the manufacturer's protocol. The colorimetric signal was measured by FLUOstar Omega plate reader (BMG Labtech).

1.2. Seahorse Assay

Cellular oxygen consumption was measured by XF96 Analyzer (Seahorse Bioscience). hPHEO1 cells were plated at a density of 40,000 cells per well and cells were allowed to grow overnight. The cells were washed by the XF based assay media supplemented with 10mM glucose, 10mM sodium pyruvate and 2mM glutamine and incubated in non-CO_2 incubator for 45 mins prior to loading into the machine. The concentrations for compounds injected during the analysis were 1.5 μM oligomycin, 2.0 μM FCCP and 0.5 μM Rot/AA. Data was obtained and analyzed using the XF96 Analyzer software.

1.3. SDHB cDNA Ectopic Expression

The cDNA expression vector of human SDHB pLYS5-SDHB-Flag was purchased from Addgene (#50055). The plasmid was transfected into hpheo1 cells by lipofectamine 3000 (Invitrogen/ThermoFisher Scientific) and incubated for 2 days. The human SDHB expression was detected by immunoblotting.

1.4. GSH/GSSG Measurement and Quantification

The GSH and GSSG level was measured using GSH/GSSG-Glo assay (Promega) according to the manufacturer's manual. Luminescence signal of total glutathione and GSSG was measured by a Polarstar Optima plate reader. The luminescence signal was normalized to protein level.

1.5. Metastatic Allograft Mouse Model

MPC *SDHB*^{WT} Luc cells (1.5 million) suspended in normal phosphate-buffered saline (PBS) solution were injected into the tail vein of female athymic mice (Ncr-nu/nu aged 8 weeks). Ten days after inoculation, animals were screened by IVIS imaging system to evaluate the tumor growth. Mice were randomized into two groups (8 mice/group) and treated with PBS and brusatol, respectively. Brusatol was injected intraperitoneal (i.p.) every other day at 1 mg/kg. All animals were carefully monitored every day and tumor growth was recorded every week by IVIS in vivo imaging system. At the end of the experiments, all animals were sacrificed, and liver metastatic lesions were harvested for further analysis.

2. Supplementary Figures

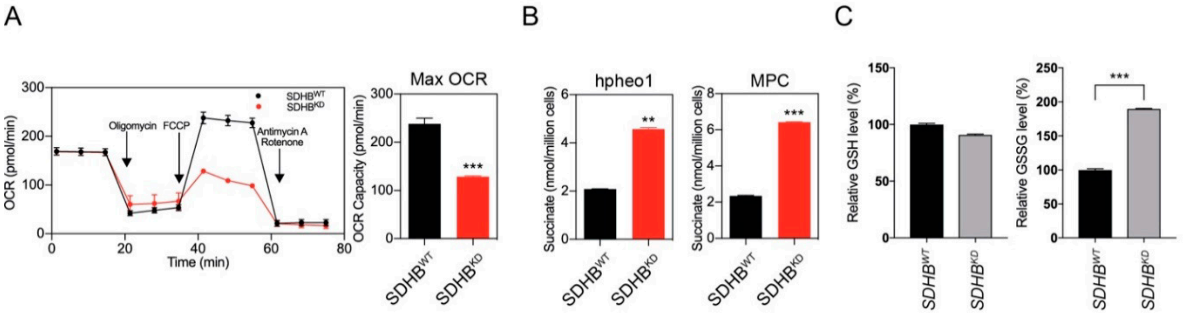

Figure S1. Metabolic characterization of *SDHB*-deficient cells. (**A**) Seahorse assay showed the oxidative metabolism in $SDHB^{WT}$ and $SDHB^{KD}$ MPC cells. OCR, oxygen consumption rate. ***$p < 0.001$. (**B**) Succinate measurement in $SDHB^{WT}$ and $SDHB^{KD}$ MPC and hpheo1 cells. **$p < 0.01$; ***$p < 0.001$. (**C**) Quantification of GSH and GSSG level from Figure. 1E. ***$p < 0.001$.

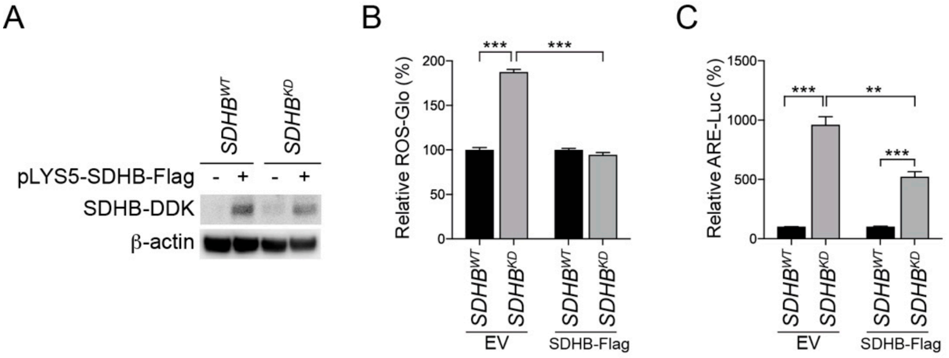

Figure S2. Ectopic expression of SDHB restored redox balance in *SDHB*-deficient cells. (**A**) Immunoblotting showed ectopic expression of SDHB in $SDHB^{WT}$ and $SDHB^{KD}$ hpheo1 cells. β-actin was used as loading control. (**B**) ROS measurement using ROS-Glo assay in $SDHB^{WT}$ and $SDHB^{KD}$ hpheo1 cells after ectopic expression of SDHB. EV, empty vector. ***$p < 0.001$. (**C**) ARE luciferase activity measurement in $SDHB^{WT}$ and $SDHB^{KD}$ hpheo1 cells after ectopic expression of SDHB. EV, empty vector. **$p < 0.01$; ***$p < 0.001$.

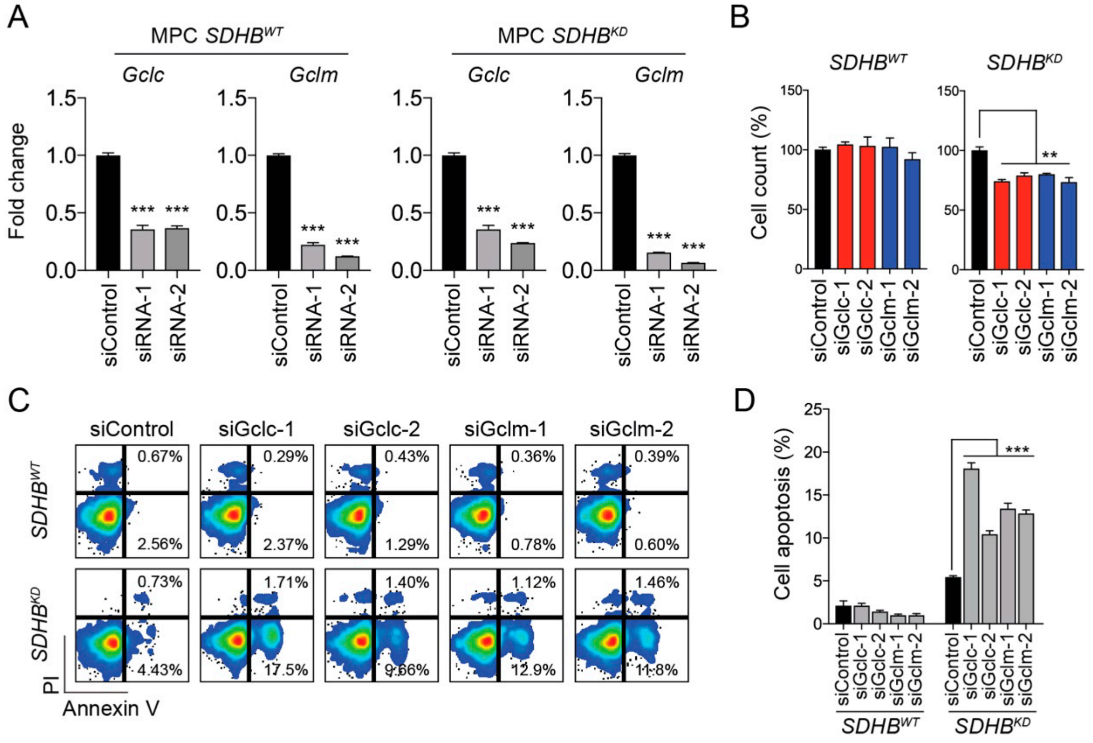

Figure S3. Glutathione synthesis is crucial for the survival in *SDHB*-deficient MPC cells. (**A**) Quantitative real-time PCR shows the knockdown efficiency of siRNA targeting *Gclc* and *Gclm* in MPC cells. $^{***}p < 0.001$. (**B**) Direct cell count shows reduced cell number of $SDHB^{KD}$ MPC cells with siRNAs targeting *Gclc and Gclm*. ** $p < 0.01$. (**C**) Annexin V/PI apoptosis assay shows the apoptotic changes of $SDHB^{KD}$ MPC cells with siRNAs targeting *Gclc and Gclm*. (**D**) Quantification of apoptotic cells in Figure. S3C. $^{***}p < 0.001$.

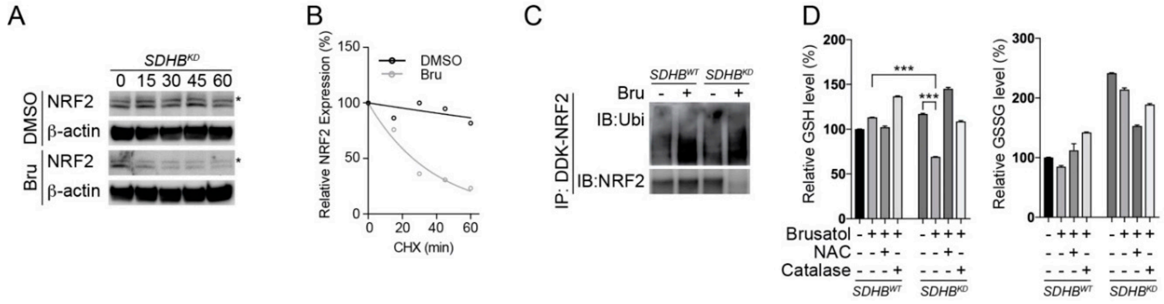

Figure S4. Brusatol induced NRF2 ubiquitination and degradation and suppressed glutathione synthesis. (**A**) CHX pulse chase assay showed that NRF2 is destabilized under brusatol treatment. β-actin was used as internal control. * represents NRF2 band. (**B**) Quantification of NRF2 half-life from Figure. S4A. (**C**) Immunoprecipitation assay shows brusatol treatment resulted in increased NRF2 ubiquitination. (**D**) Quantification of GSH and GSSG level from Figure. 4D. $^{***}p < 0.001$.

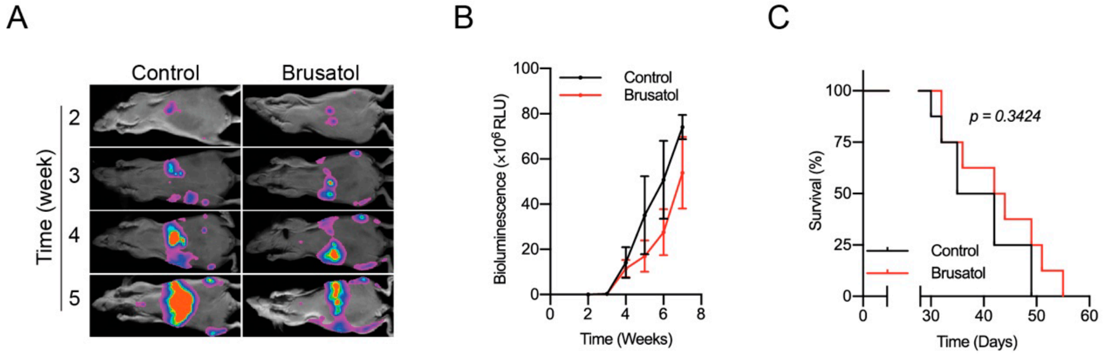

Figure S5. Brusatol treatment in SDHB wild-type MPC allograft in vivo. (**A**) Luciferase imaging shows hepatic lesions of *SDHB*^{WT} MPC cells in vivo. (**B**) Quantification of tumor volume shown in Figure. S5A. (**C**) Kaplan–Meier analysis shows overall survival of *SDHB*^{WT} tumor-bearing animal (p = 0.3424).

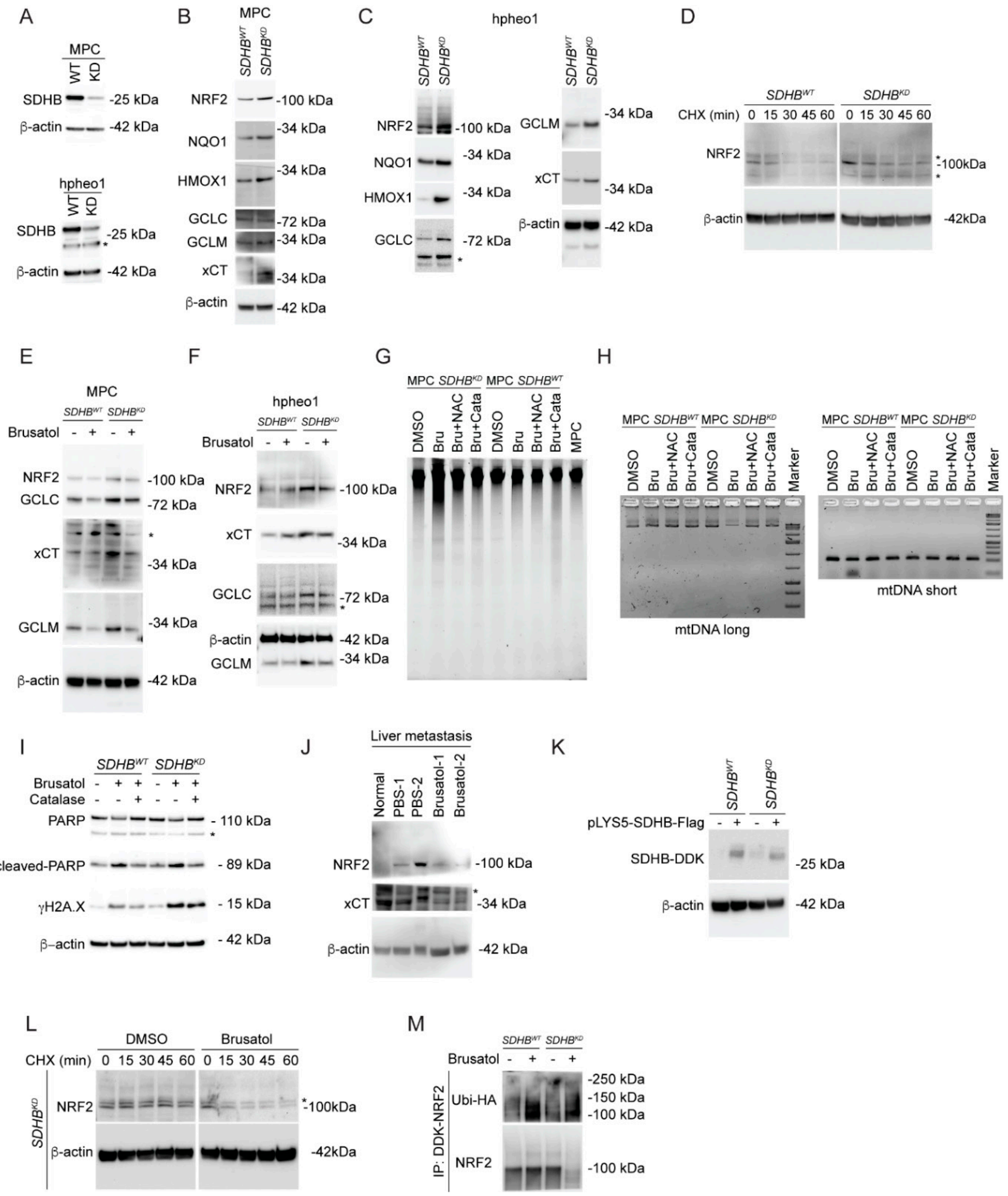

Figure S6. Western Blot and DNA electrophoresis images**.** (**A**) Images of Figure 1A. (**B**) Images of Figure 2C MPC cells. (**C**) Images of Figure 2C hpheo1 cells. (**D**) Images of Figure 2D. (**E**) Images of Figure 4B MPC cells. (**F**) Images of Figure 4B hpheo1 cells. (**G**) Images of Figure 5D. (**H**) Images of Figure 5E. (**I**) Images of Figure 6F. (**J**) Images of Figure 7E. (**K**) Images of Fig. S2A. (**L**) Images of Fig. S4A. (**M**) Images of Fig. S4C. * shows nonspecific band.

© 2020 by the authors. Licensee MDPI, Basel, Switzerland. This article is an open access article distributed under the terms and conditions of the Creative Commons Attribution (CC BY) license (http://creativecommons.org/licenses/by/4.0/).
